# HLAProfiler utilizes *k*-mer profiles to improve HLA calling accuracy for rare and common alleles in RNA-seq data

**DOI:** 10.1186/s13073-017-0473-6

**Published:** 2017-09-27

**Authors:** Martin L. Buchkovich, Chad C. Brown, Kimberly Robasky, Shengjie Chai, Sharon Westfall, Benjamin G. Vincent, Eric T. Weimer, Jason G. Powers

**Affiliations:** 1 Translational Genomics Department, Q2 Solutions | EA Genomics, a Quintiles Quest Joint Venture, Morrisville, NC 27560 USA; 2 Lineberger Comprehensive Cancer Center, University of North Carolina at Chapel Hill, Chapel Hill, NC 27599 USA; 3 Curriculum in Bioinformatics and Computational Biology, University of North Carolina at Chapel Hill, Chapel Hill, NC 27599 USA; 4 Division of Hematology/Oncology, Department of Medicine, University of North Carolina at Chapel Hill, Chapel Hill, NC 27599 USA; 5 Department of Pathology and Laboratory Medicine, University of North Carolina at Chapel Hill, Chapel Hill, NC 27599 USA

**Keywords:** HLA, HSCT, Transplantation, Immunology, RNA-sequencing

## Abstract

**Background:**

The human leukocyte antigen (HLA) system is a genomic region involved in regulating the human immune system by encoding cell membrane major histocompatibility complex (MHC) proteins that are responsible for self-recognition. Understanding the variation in this region provides important insights into autoimmune disorders, disease susceptibility, oncological immunotherapy, regenerative medicine, transplant rejection, and toxicogenomics. Traditional approaches to HLA typing are low throughput, target only a few genes, are labor intensive and costly, or require specialized protocols. RNA sequencing promises a relatively inexpensive, high-throughput solution for HLA calling across all genes, with the bonus of complete transcriptome information and widespread availability of historical data. Existing tools have been limited in their ability to accurately and comprehensively call HLA genes from RNA-seq data.

**Results:**

We created HLAProfiler (https://github.com/ExpressionAnalysis/HLAProfiler), a *k*-mer profile-based method for HLA calling in RNA-seq data which can identify rare and common HLA alleles with > 99% accuracy at two-field precision in both biological and simulated data. For 68% of novel alleles not present in the reference database, HLAProfiler can correctly identify the two-field precision or exact coding sequence, a significant advance over existing algorithms.

**Conclusions:**

HLAProfiler allows for accurate HLA calls in RNA-seq data, reliably expanding the utility of these data in HLA-related research and enabling advances across a broad range of disciplines. Additionally, by using the observed data to identify potential novel alleles and update partial alleles, HLAProfiler will facilitate further improvements to the existing database of reference HLA alleles. HLAProfiler is available at https://expressionanalysis.github.io/HLAProfiler/.

**Electronic supplementary material:**

The online version of this article (doi:10.1186/s13073-017-0473-6) contains supplementary material, which is available to authorized users.

## Background

The human leukocyte antigen (HLA) complex is the set of genes on chromosome 6 encoding proteins of the major histocompatibility complex (MHC). These genes are divided into multiple classes with similar but distinct functions. Class I genes, such as HLA-A, HLA-B, and HLA-C, are expressed in nearly all nucleated cells and are important for recognizing endogenous foreign antigens. These antigens can arise via infection or from somatic variations, such as those introduced in cancer. Class II genes, expressed on antigen-presenting cells, generally recognize exogenous foreign antigens, such as viral peptides entering the cytoplasm after apoptosis of infected cells. Other genes in the HLA complex can have more specialized functions (e.g., HLA-E for cell recognition by natural killer cells) or are associated with HLA (e.g., TAP1/TAP2 for antigen peptide transport) [1, 2].

HLA genes are highly polymorphic, and the number of known alleles continues to grow [3]. Accurately identifying which alleles are present in an individual is important in many areas, such as drug safety [4], disease susceptibility [5, 6], neoantigen prediction for cancer treatment [7], regenerative medicine [8], and organ transplantation [9]. Traditional “gold standard” assays for HLA typing, such as sequence-specific oligonucleotide probe PCR (PCR-SSOP), sequence-specific primed PCR (PCR-SSP), and Sanger-based sequence-based typing (SBT), are labor intensive, costly, and relatively low throughput [10, 11].

A new class of assays has emerged in recent years which uses targeted next-generation sequencing (NGS)-based methods as a new gold standard while increasing throughput and decreasing both the reagent and labor costs of HLA typing [12].

Most of these NGS assays for HLA typing use customized PCR, designed to specifically interrogate the HLA region, such as TruSight HLA (Illumina), Holotype (Omixon), and NGSgo (GenDx). Published data suggest that these platforms are highly accurate for calling HLA types [12, 13]. Due to their targeted nature, however, these assays do not provide information outside the HLA region, preventing the identification of other biologically meaningful data such as somatic variation found in other genes, thereby limiting their general utility. Also, only a limited amount of HLA-specific targeted-capture sequencing data is publicly available, making HLA typing of historical data using these methods difficult. To overcome the limitations inherent to HLA targeted DNA approaches, methods have been developed to identify HLA types in whole-genome sequencing (WGS) and whole-exome sequencing (WES) data [14, 15], but these methods also have limitations.

Due to the high variability found in the HLA loci, these regions must have sufficient coverage to accurately ascertain HLA types. Both studies found that it is possible to call HLA types accurately from WGS; however, both also found that this high accuracy requires nearly half a billion sequencing reads [14]. Although sequencing costs continue to drop, this depth of sequencing is currently not a practical way to resolve the thousands of possible HLA alleles for many investigators [14]. Conversely, while WES and other large targeted panels require far fewer sequencing reads to achieve a high depth of coverage, the ability to correctly identify HLA types using general targeted DNA-capture methods is highly dependent on the sequences of the probes used to capture the DNA. Polymorphisms within HLA genes may disrupt capture and cause portions of or entire exons to be missing from the sequencing data. This can be especially true if rare alleles or as-yet-undiscovered alleles were not considered during probe design. Finally, heterozygous alleles may have different capture efficiencies, leading to non-equal sequencing coverage, potentially complicating analysis. Indeed, HLA-typing accuracy for exome sequencing has been shown to be poor [15, 16].

Based on these limitations, HLA typing in RNA sequencing (RNA-seq) data promises several advantages: i) it provides an unbiased dataset that fully covers both fully expressed parental alleles equally; ii) no special reagents or protocols are needed; and iii) RNA-seq data offer a myriad of uses beyond HLA typing. Existing tools have been used to perform HLA typing using RNA-seq data with limited success, suffering from poor accuracy or the inability to call rare or novel alleles or offering HLA calling for only a limited number of genes.

To overcome these limitations, we have developed HLAProfiler to accurately determine HLA types using RNA-seq data. HLAProfiler uses the *k*-mer content of RNA-seq reads to identify the most likely HLA type for the sample. Using both simulated RNA-seq data and RNA-seq data from lymphoblastoid cell lines, we have evaluated the performance of HLAProfiler. In data simulating both common and rare alleles, HLAProfiler correctly identified > 99% of alleles. In the biological dataset, HLAProfiler correctly identified HLA alleles with > 98% concordance to gold standard Sanger sequencing calls. After using a third technology to resolve the discrepancies with Sanger sequencing, HLAProfiler accuracy increased to > 99% in the biological data. We identified multiple cases where HLAProfiler correctly identified alleles associated with disease risks that Sanger missed, including ankylosing spondylitis (AS). Additionally, HLAProfiler has the ability to correctly identify the full protein sequences of both novel alleles and alleles with an incomplete reference sequence. Finally, we demonstrate that HLAProfiler can be extended to identify the alleles of other immunologically important gene classes, such as killer-cell immunoglobulin-like receptor (KIR) genes. Overall, we demonstrate that HLAProfiler excels at correctly identifying HLA alleles in the wealth of RNA-seq data already generated and being generated daily, opening the door for further advances in immunological and disease research.

## Implementation

To assess the accuracy of HLAProfiler and five other HLA-typing software tools, two sets of data were used. The first was a set of 358 real samples that were HLA typed using Sanger sequencing and whose RNA-seq data were also publicly available. The second set comprised data simulated to assess the accuracy of each tool for common, rare, and novel alleles, as well as KIR genes.

### RNA-seq data from lymphoblastoid cell lines from the Geuvadis study

Publicly available RNA-seq data from the Geuvadis study were used [17]. Briefly, 462 lymphoblastoid cell lines (LCLs) were sequenced on the Illumina HiSeq2000 platform using paired-end 75-bp reads, with 29.9 million reads on average (range 8.6–83 million, with an interquartile range of 24–34 million). HLA types for 358 of these samples from diverse populations (Additional file 1: Table S1) were determined using Sanger sequencing for HLA-A, HLA-B, HLA-C, HLA-DRB1, and HLA-DQB1 [18, 19]. Sanger sequencing uses select exons to assign an HLA type, introducing ambiguity into the gold standard HLA calls. To account for this ambiguity, we downloaded the curated set of ambiguous HLA alleles from IMGT/IPD [3] and converted all predicted and gold standard HLA calls to the “G” group, when applicable, before calculating concordance. To assess the accuracy of HLA calling programs under reduced read numbers, this dataset was also downsampled to five million paired reads.

### Simulated RNA-seq data

Five different types of simulated datasets were created. In all cases, a transcript FASTA file was generated from the human transcriptome extracted from GENCODE v24 [20, 21], with the HLA genes replaced with specific HLA alleles unique to each subject. HLA allele sequences were obtained from the IMGT/HLA database (v24) [22]. Each sequence was over-represented by repeating it 100 times in the FASTA file so that the total number of HLA reads roughly matched those observed in the Geuvadis study. This modified transcript file was then used to generate simulated FASTQ files using the program wgsim. Specifically, 20 million paired-end 50-bp reads were generated for each individual, using an error rate of 0.001 (base qualities of Q30), a mean insert size of 250, and an insert standard deviation of 75. Wgsim did not allow for the simulation of genes with sequence lengths less than 400 with the current parameter set. Although this restriction did not affect HLA or KIR alleles, it did affect many other real human transcripts, potentially making the simulated data less diverse than a truly biological sample. For this reason, wgsim was altered slightly to allow for simulation of genes with sequence lengths down to 200 bp using the current parameter set. All simulated datasets are available at http://www.q2labsolutions.com/genomics-laboratories/bioinformatics (select “Request Example Data”).

The first dataset simulated RNA-seq data from 109 subjects with HLA types made to be consistent with the two-field HLA types for samples from the GeT-RM program hosted by the Centers for Disease Control [23, 24]. For the alleles where two-field precision matches several actual HLA alleles (e.g., A*01:01, matches A*01:01:01:01, A*01:01:01:02, etc.), the first allele with a “complete” status (i.e., lowest accession number and the entire transcript sequence is known) from IMGT was chosen. Most of the simulated HLA alleles for this dataset were previously categorized as “common” or “well documented” [25, 26].

The second dataset simulated rare alleles. This set was generated using alleles with complete status (entire sequence known) from IMGT that were not previously listed as “common” or “well documented” (Additional file 1: Table S2). For this set, HLA types for 100 individuals (200 alleles) were simulated by randomly drawing from these rare alleles with replacement.

Historically, only exons 2 and 3 of class I genes or exon 2 of class II genes have been submitted to the IMGT/HLA database, because these exons contain the variability influencing peptide binding [3]. As a result, over 87% of the alleles listed in the IMGT/HLA database have partial status, where the entire sequence has not been submitted. Typing for these alleles is challenging since many reads will match much better to similar alleles whose complete sequence is in the database than to the incomplete, correct sequence. To simulate this unique situation, the third simulated dataset was identical to the second dataset except that each sample had one allele swapped with an allele that had a partial, or incomplete, sequence in IMGT/HLA release 3.24.0 but later had a complete sequence in release 3.26.0 (Additional file 1: Table S2). In these cases, the full allele sequences from the later release were used to simulate the data, but information from only the earlier, incomplete release was included in the profile database used for calling HLA types.

The fourth simulation dataset includes novel alleles (i.e., not in the IMGT/HLA database) and was created similarly to the third simulation dataset. Alleles included in release 3.26.0 with a complete sequence but absent in 3.24.0 were considered novel alleles. The novel allele dataset was generated by taking the “rare allele” dataset and replacing exactly one allele for each individual with a novel allele (Additional file 1: Table S2).

The fifth simulated dataset was for alleles from the KIR genes [27]. For this simulation, full alleles from the IMGT/KIR database were used to generate two alleles randomly for each of the 17 different KIR genes across ten samples. The simulation parameters were the same as for the other datasets.

### HLAProfiler algorithm overview

**Fig. 1:**
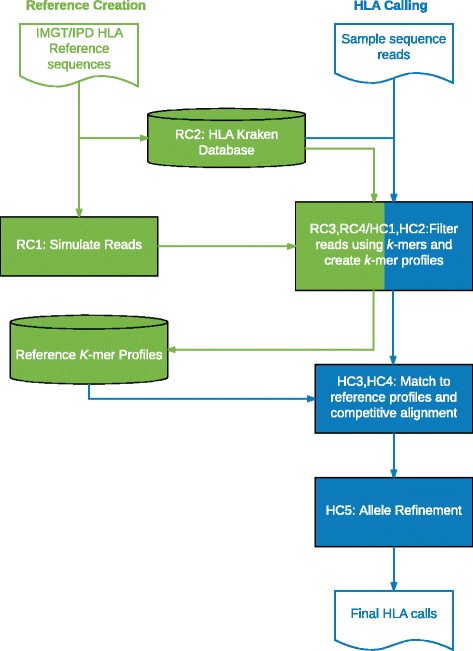
Overview of the HLAProfiler workflow. The HLAProfiler workflows to create the reference *k*-mer profile database (*green*) and HLA calling in RNA-seq data (*blue*). Each step label in the workflow corresponds to the text (see “Implementation”). The workflows share the *k*-mer filtering and profile creation step (*blue*/*green box*)

The constantly increasing number of reference HLA alleles makes HLA calling in RNA-seq data a daunting task that can more easily be accomplished by reducing the problem’s search space. Rather than using a series of traditional alignment and/or contig assembly steps for this reduction, HLAProfiler relies on a series of *k*-mer-based steps. A difficult and time-consuming first step of HLA calling in RNA-seq data is identifying the small fraction of reads originating from the HLA region and the specific gene from which they originate. While this filtering is often accomplished with sequence alignment, HLAProfiler takes advantage of a taxonomic sequence classification tool, Kraken [28], to quickly identify FASTQ reads uniquely arising from a particular HLA gene. Using the *k*-mer composition of each read pair, and a taxonomic reference that has been pre-built from the IPD-IGMT/HLA database of sequences, this step can be accomplished very quickly and accurately. Having reduced the observed data to reads containing information specific to HLA genes, the next problem is reducing the vast number of alleles for each gene to the most likely candidates. By breaking apart paired reads and examining their *k*-mer content separately, each set of gene-specific reads can be quickly reduced to an “observed *k-*mer” profile for the sample. In a quick and simple filtering step, HLAProfiler compares the *k*-mers in the observed *k*-mer profile to a reference set of allele profiles, reducing the number of candidate alleles from up to many thousands to just a few hundred (default 100). A subsequent, more complex step comparing the observed profile to all possible candidate pairs identifies the most likely candidate pairs (default 20). The final HLA call is decided using a combined score from the *k*-mer profile comparison and a simple alignment of the paired-end reads to the final candidate alleles. These *k-*mer-based methods greatly reduce the time required for this paired-end alignment step, and allows for *k-*mer profile comparison. Together these two steps provide complementary evidence of the correct HLA call. Below, we describe in more detail the algorithms to a) make the reference and b) call HLA alleles for a sample (Fig. 1).

### Creating a reference for HLAProfiler

The reference HLA *k*-mer profiles were constructed in four steps: reference profile creation step (RC)1) simulate FASTQ reads using a reference FASTA file of HLA sequences; RC2) create a Kraken database using a custom HLA taxonomy; RC3) use Kraken to assign the simulated reads to HLA genes in the taxonomy; and RC4) aggregate the *k*-mer counts for each allele into gene-level, reference *k*-mer profiles. Once created, the reference profiles can be reused to identify types in various new RNA-seq samples. HLAProfiler has a build option to build a reference database. Because reads are simulated for every allele in the database (>14K alleles), database creation can take as long as 24 h using 48 cores. The database used in this study was created using the complete IPD-IMGT/HLA version 3.24.0 reference. Database creation steps are detailed below.

#### RC1: simulate FASTQ using a reference FASTA of HLA sequences

The gene-level reference profiles are created using an HLA reference nucleotide FASTA file following the standard IPD-IMGT/HLA nomenclature. While the work described here uses the nucleotide FASTA from IPD-IMGT/HLA release 3.24.0, reference profile creation will also work with custom reference sequences as long as the same naming conventions are followed.

A simulated FASTQ file is made from each unique allele sequence in the reference FASTA. Identical allele sequences are merged together, and the merge is reported any time that sequence is included among the predicted HLA types. The paired-end read fragment lengths are simulated assuming a Pareto distribution, with a user-defined scale, shape, and maximum insert size. Additionally, the user specifies read length and number of reads. Reads simulated for this work used the following default parameters: scale 80, shape 0.7, max_insert 1000, num_reads 500,000, read_length 50. For all simulations, read 1 is reverse complemented, comparable to a stranded sequencing protocol.

#### RC2: create a Kraken database using a custom HLA taxonomy

A custom HLA taxonomy is created from the same reference FASTA used for the simulation of allele reads (Additional file 1: Figure S1). The taxonomic organization is based on the standard HLA nomenclature. The tree has two main branches: HLA alleles and the distractome. The distractome comprises all gene transcripts except transcripts falling within the HLA region and is important for accounting for homology with non-HLA genes. The HLA common ancestor has three branches: class I genes, class II genes, and other HLA genes. Leaves of the tree on these branches represent each individual allele (i.e., A*02:02:01:01), with all other nodes, or common ancestors, on each branch representing the various fields of precision for that leaf (i.e., A, A*02, A*02:02, A*02:02:01). The distractome was created using transcripts from GENCODE version 24. This custom taxonomy was built into a Kraken database using the default HLAProfiler parameters for minimizer length (-mi 3) and *k*-mer length (-k 31) using 24 threads (-c 24). The taxonomy uses the NCBI database format with node names and node relationships found in names.dmp and nodes.dmp, respectively, located in the taxonomy subdirectory of the built database.

#### RC3: use Kraken to assign the simulated reads to HLA genes

Kraken is typically used to understand species diversity in sequencing data, assuming a given taxonomy. This methodology was applied to HLAProfiler using only the HLA taxonomy rather than the standard NCBI species taxonomy. Leveraging the *k*-mers unique to each taxonomic unit, Kraken outputs a taxonomic classification for each sequence read. For HLA typing, we have modified Kraken to split the reads into individual FASTQ files based on these classifications [29]. For HLAProfiler, reads are placed into FASTQ files corresponding to the gene level, or higher, node in the tree.

#### RC4: aggregate the k-mer counts for each allele into gene-level profiles

All observed *k*-mers of length 50 are counted independently for each HLA gene-specific FASTQ file and collected across all alleles for a particular gene to create a list of *k*-mer hash table counts. This same procedure is used for calling HLA types, where the profile of the *k*-mers observed in the sample FASTQ is calculated and can be compared to the *k*-mer profile for each allele of the gene. The *k*-mer classification approach of Kraken, combined with the custom taxonomy helps to overcome homology between HLA genes. While *k*-mers unique to each gene enable Kraken to correctly classify the read, Kraken also considers the lowest common ancestors when making a classification. A read with many *k*-mers shared across genes and just a few unique *k-*mers can still be filtered to the proper gene. Kraken also processes paired-end reads together, further increasing the likelihood of gene-specific *k*-mers and successful classification.

### Calling HLA alleles using HLAProfiler and an HLAProfiler-specific reference

Using the HLA *k-*mer profiles to type samples involves five steps: HC1) use Kraken to assign sample FASTQ reads to HLA genes; HC2) aggregate the *k*-mer counts for each allele into gene-level sample profiles; HC3) compare sample *k*-mer count profiles to the reference profiles to determine top allele pair candidates; HC4) competitively align each pair of candidate alleles and count the number of reads aligning uniquely to only one of the pairs; and HC5) refine the allele call to identify novel or updated alleles. The final score of the predicted HLA type is based on the strength of the sample *k*-mer profile match to the reference profile and the competitive pairwise alignment counts.

#### HC1: use Kraken to assign sample FASTQ reads to HLA genes

See “RC3: use Kraken to assign the simulated reads to HLA genes”.

#### HC2: aggregate the *k*-mer counts for each allele into gene-level sample profiles

See “RC4: Aggregate the *k*-mer counts for each allele into gene-level reference profiles”.

#### HC3: compare sample *k*-mer count profiles to reference profiles to determine top allele pair candidates

If a particular *k-*mer from the profile of a candidate HLA allele is found in the observed reads, the *k*-mer is considered accounted for. To reduce the negative influences of sequencing errors, infrequently (relative to overall sequence depth) observed *k*-mers are not considered as part of the observed profile. The counts of all *k*-mers in the profile that are accounted for are divided by the total count of *k*-mers in the profile to calculate the fraction of the profile observed. The top *n* semifinalists (default *n =* 100) with the highest proportion are carried into downstream analyses. In cases where allele *n* + 1 and *n* are tied, the number of semifinalists (*n*) is increased until the score for *n* + 1 is less than *n.* For each of the resulting *n* × *n* pairwise combinations, the proportion of observed FASTQ *k*-mers accounted for by the combined profile (*PropReads*) and the proportion of the reference profile *k*-mers accounted for by the observed read *k-*mers (*PropProf*) are calculated. *K*-mers from observed reads with one or fewer counts are not considered in either calculation. In addition, the Pearson correlation (*Cor*) between the log of the profile and the log of the read *k*-mers is calculated. Error is calculated as:

$$ Error=\left(1- PropProf\right)+\left(1- PropReads\right)+\left(1- Cor\right) $$

and the top *m* finalist (default *m* = 20) allele pair candidates are retained for competitive alignment. A reference *k-*mer profile contains both the *k*-mers expected from an allele and the expected proportion of each k-mer in the profile. While the correlation (*Cor*) is influenced by both *k-*mer composition and *k-*mer counts of the observed profile, the metrics *PropReads* and *PropProf* are solely influenced by the *k-*mer composition. HLAProfiler does not test for or estimate allelic expression of HLA alleles, but the inclusion of *PropReads* and *PropProf* helps to mitigate negative impacts of expression bias on HLA calling.

#### HC4: competitive alignment between the top 20 candidate allele pairs

An *m* × *m* alignment score matrix (ASM) is initialized to zero. Each of the original FASTQ read pairs is then checked for an exact match to each of the top 20 candidate allele pairs. If both reads in the pair match one of the alleles from candidate pair i, but zero alleles from candidate pair j, the (i, j)th entry of the ASM is incremented by a quality-determined score *S*:

$$ S=\kern0.6em \left\{\begin{array}{c}0\kern0.72em if\;Q\le {Q}_{min}\\ {}\frac{Q-{Q}_{min}}{\;35-{Q}_{min}}\; if\;{Q}_{min}<Q<35\\ {}1\kern0.6em if\;Q\ge 35\end{array}\right. $$

where *Q* is the lowest quality score of one of the bases in either read 1 or read 2 and *Q*_*min*_ is the minimum quality threshold (default 20). This process is repeated for all reads across all possible pairwise combinations of candidate allele pairs. The resulting row sums represent the quality-weighted count read unique to the allele pair in the row, while the column sums represent the weighted counts of reads unique to other alleles when compared to the column allele pair. Compared to other allele pairs, the correct pair is expected to have more unique reads resulting in a higher row sum and lower column sum. In some cases, particularly with a low number of unique reads, sequencing error can artificially increase the column sum. Each entry of the ASM is incremented by the total number of matching reads divided by 10,000 to help protect against these situations where the noise (e.g., unique matches due to sequencing error) outweighs the signal (i.e., unique matches due to real differences in alleles). This adjustment favors allele pairs with a higher row sum. For example, if the correct allele pair in a matrix of four pairs has a ratio of 7/4 (row to column) and the incorrect allele has a ratio of 4/2, after a total adjustment to the sums of 3.75 (1.25 per cell), the correct allele will now have a higher ratio (10.75/7.75 compared to 7.75/5.75). An HLA score for each read pair is calculated using:

$$ Score(i)={\left(\frac{sum\left( ASM\left[i\right]\right)}{sum\left( ASM\left[,i\right]\right)}\right)}^{P\ast}\;\frac{1}{Error(i)} $$

where *P*, ranging from 0 to 1, is the power assigned to this competitive alignment score (default *P* = 0.25). The top-scoring allele pair is then selected as the predicted allele pair.

#### HC5: allele refinement

This step is optional. Paired reads are mapped using a custom aligner a final time to the predicted allele pair, this time allowing for one mismatch. During this process, the read support for each possible nucleotide is recorded. Positions that have more support for a mismatching nucleotide than for the reference nucleotide (default > 75% of coverage mismatch) are substituted into a putative novel allele sequence. All reference alleles for the same gene from IMGT are then checked to determine whether they are an exact substring of the novel allele and are also listed as having only a “partial” sequence with one or more missing exons. If any allele satisfies these requirements, the predicted allele is changed to the partial reference allele, with a “U” (for updated) added to the accession ID. If no alleles satisfy these requirements, the accession ID of the top-scoring candidate is appended with an “N” (for novel). In either case, the modified sequence is output to file by the program, and a new simulation profile is created for the novel allele. If the allele refinement option is specified as “recalculate” or “all”, then all metrics are calculated for the novel allele to obtain an updated prediction score.

### Considerations of *k*-mer size

HLAProfiler utilizes *k-*mers in two different ways: 1) sequence read filtering; and 2) profile creation. The longer the *k-*mer, the more likely it is to be unique to a specific HLA gene or allele. Using shorter *k*-mer sizes will decrease the number of unique *k-*mers across the HLA genes, which in turn will decrease the memory footprint of the Kraken database, but also reduce the sensitivity of filtering. We recommend using the maximum *k*-mer size allowed by Kraken of 31 bp.

The fewer the number of HLA alleles that contain a given *k*-mer, the greater the contribution of that *k*-mer for identifying the correct HLA alleles. As a profile captures the combination and quantity of *k*-mers of each allele, even *k*-mers shared across many alleles can contribute valuable information. The *k-*mer size is mainly limited by sequence read length, as HLAProfiler will not work properly if *k* is greater than the sequence length. While there is no lower bound on *k*, small values for *k* will negatively impact algorithm performance. Additionally, using a *k* that is much smaller than the sequence length can increase runtime of HLAProfiler. A final consideration is that each value for *k* requires the creation of a new set of reference *k-*mer profiles, a computationally intense process. Given these considerations, we have characterized HLAProfiler performance using a *k-*mer size of 50, which maximizes *k-*mer content of the profiles while accommodating many of the current and historic RNA-seq read lengths.

### TruSight HLA confirmation to update gold standard truth

A total of 324 Geuvadis samples were concordant between Sanger sequencing, RNA-seq with HLAProfiler, and RNA-seq with OptiType (when available) for all five reported genes (HLA-A, HLA-B, HLA-C, DRB1, and DQB1). For these, we assumed that this reflected the true state of the sample. For the 34 samples that had at least one allele from any gene that was discordant between Sanger sequencing and either OptiType or HLAProfiler, discrepancies were resolved by further sequencing using Illumina’s TruSight HLA assay. Two samples with only one-field accuracy reported by Sanger sequencing were also tested, In all cases, the TruSight HLA result matched either one of the RNA-seq results or the Sanger result.

Whenever TruSight HLA represented a consensus (matching either Sanger or RNA-seq), we assumed that the consensus type represents the true state. This information was used in the establishment of an updated truth for the Geuvadis data, from which accuracy, not just concordance to another technology, can be estimated. The results of TruSight HLA are available at http://www.q2labsolutions.com/genomics-laboratories/bioinformatics (select “Request Example Data”).

### HLAProfiler software

HLAProfiler is written entirely in Perl, with the exception of Jellyfish [30], a Kraken dependency, and the modified version of Kraken. All analyses were performed on a Linux cluster with eight CPU cores and 8 GB of memory. Computational performance was assessed from a random sample of 30 Geuvadis FASTQ pairs using 8 GB of RAM and 12 cores of a Dell PowerEdge R720 server with 256 GB of total RAM and two 12-core CPUs. Total runtimes ranged from 7 to 17 min, with a mean of 12.5 min, making HLAProfiler’s speed comparable to the other available tools (Additional file 1: Figure S2). These analyses used HLAProfiler v1.0.0. The latest version of HLAProfiler can be downloaded from GitHub (see “Availability of data and materials”) or as part of the bioconda repository (https://bioconda.github.io/recipes/hlaprofiler/README.html).

### Running other callers

All callers were run on a Linux cluster using eight CPU cores. OptiType [31] was installed as directed and run using razers3-3.5.3 and glpk and default parameters. We ran seq2HLA v2.2 [32] against a reference using only the “common” and “well-documented” alleles and default parameters. HLAForest [33] was installed as directed, and a new reference was created using IMGT/HLA v3.24.0 sequences. CallHaplotypesPE.sh was run using default parameters to type each sample. HLAMiner [16] was installed as directed and run using default parameters and a reference database. Phlat-1.0 [34] used IMGT/HLA version 3.8.0 and Bowtie2 version 2.3.0. The paired-end parameter “-pe” was set to 1, under which the data are treated as paired-end. Orientation parameter “-orientation” was set to “—fr”, under which the orientation of the mate pair reads are in forward and reverse. Where documentation existed, every attempt was made to update the reference for these tools to be comparable to IMGTv3.24.0. HLAForest and HLAMiner were successfully updated, but HLAMiner performance decreased with the updated reference, so the reference packaged with the software was used.

## Results

HLA types were predicted in five simulated RNA-seq datasets and from biological RNA-seq data from lymphoblastoid cells using HLAProfiler. For comparison, types were also predicted using five other tools capable of detecting HLA types in RNA-seq data: OptiType, PHLAT, seq2hla, HLAMiner, and HLAForest. The accuracy of calls from simulated data was calculated as the proportion of alleles that matched exactly to the truth at the specified precision. Likewise, accuracy for the biological data was calculated by comparing the allele calls to the Sanger sequencing/TruSight HLA consensus described in “Implementation”. One-field precision refers to the allele group (results not shown), two-field precision refers to the protein sequence, and exact precision refers to an exact match of the allele at the highest precision possible (some alleles have only two-field precision defined). Allele calls unable to be predicted by a tool in samples with truth information available for the gene were considered incorrect or discordant.

### HLAProfiler perfectly called HLA types in RNA-seq data simulated from GeT-RM HLA types

**Fig. 2:**
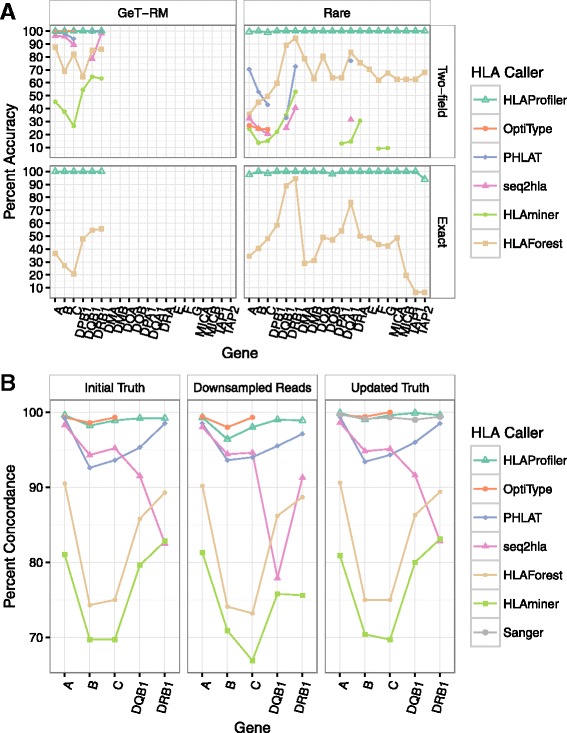
HLA calling accuracy. **a** The accuracy of HLA calling was evaluated for six algorithms. Datasets were simulated using GeT-RM alleles from 109 samples (*left panels*) and rare alleles for 100 samples (*right panels*) at two-field precision (*upper panels*) and exact precision (*lower panels*) when available. **b** Concordance of HLA calling in 358 lymphoblastoid cell lines compared with gold standard HLA allele calls generated by Sanger sequencing (*left panel*). Sequences were downsampled to five million reads, HLA alleles were called, and concordance was recalculated (*middle panel*). Discrepancies between HLAProfiler, OptiType, and Sanger sequencing were resolved using TruSight HLA for 38 samples, the gold standard calls were updated with the resolved genotype, and concordance was recalculated for all methods with the addition of the original Sanger sequencing calls (*right panel*)

We first calculated accuracy results for allele calls from RNA-seq data simulated using HLA types from 109 samples in the CDC’s GetRM program. Using HLA types from real individuals ensured that our initial simulation evaluated biologically relevant allele pairs. HLAProfiler had 100% accuracy at both two-field and exact-allele precision (Fig. 2a; Additional file 2: Table S3). OptiType also performed near perfectly, with two-field precision accuracy ≥ 99.5% for HLA-A, HLA-B, and HLA-C. At the time of analysis, OptiType did not call class II alleles nor did it provide exact-allele accuracy. Other callers had a wide range of accuracies but did not perform as well as HLAProfiler or OptiType at two-field precision. Only HLAForest also provided exact-allele precision but performed much worse than HLAProfiler, with accuracies less than 56% (Additional file 2: Table S3).

### HLAProfiler accurately predicted the HLA types of rare alleles

We observed that the only allele missed by OptiType in the GeT-RM simulation was a rare allele. This motivated an effort to quantify the ability of each caller to correctly identify rare alleles. We calculated the call rate and accuracy of HLA typing in 100 simulated RNA-seq datasets comprising only rare alleles. HLAProfiler had > 99.5% two-field precision accuracy and > 94.0% exact-allele accuracy for every gene, while other callers performed poorly (Fig. 2a; Additional file 2: Table S3). Indeed, with the exception of HLAForest, all callers had two-field accuracies less than 78%, and for HLAForest, accuracies ranged from 35–95%. Whether this drop in performance for rare alleles is caused by the algorithm or the use of outdated references distributed with the other tools remains unclear.

### HLAProfiler demonstrated high concordance with orthogonal methods in lymphoblastoid cell lines

Having evaluated performance in two simulated datasets, we next predicted HLA types in RNA-seq data generated by the Geuvadis consortium. These RNA-seq data, generated from 358 different LCLs, have gold standard HLA types available from orthogonal methods for genes A, B, C, DRB1, and DQB1 [18]. Once again, OptiType and HLAProfiler performed similarly on class I genes, with 99.0 and 98.9% average concordance, respectively, and > 98% concordance across all individual genes. This result is a marked improvement over other callers, which had < 96% average concordance for class I alleles (Fig. 2b; Additional file 2: Table S4; Additional file 2: Table S5). HLAProfiler also had an average concordance of 99.1% for class II genes, with 99.2% for DRB1 and 98.9% for DQB1. To evaluate HLA typing performance in samples with a low number of reads, we downsampled all 358 RNA-seq datasets to five million reads and predicted HLA types (Fig. 2b; Additional file 2: Table S4). Reduced read depths had little influence on HLAProfiler predictions for HLA-A, DRB1, and DQB1 (0.1–0.3% lower), while genes B and C were more affected, with 1.8 and 1% lower concordances, respectively. For comparison, read depth had less influence on OptiType performance, with a 0.1% increase for HLA-A and a 0.6% decrease for HLA-B and no change for HLA-C. The number of filtered, HLA gene-specific reads depends on HLA gene expression and can vary between samples and tissue types. To understand the influence of filtered read depth on accuracy, we selected at random 50 samples from the Geuvadis dataset for which all alleles were correctly predicted. After downsampling these filtered reads, we found that HLAProfiler can identify HLA type for all genes with greater than 90% accuracy with as few as 1000 reads (Additional file 2: Table S3). For comparison the number of filtered reads range from 3–100K for the full dataset from these Geuvadis samples (total read depths vary). Accuracy varies across genes for a specific read depth and factors such as the sample’s tissue type and the HLA genes of interest should be considered carefully when setting minimum read depth thresholds for HLA calling.

Multiple factors can influence HLA calling, which makes accurate HLA identification for every gene across every sample extremely difficult when using RNA-seq data. Because accurate HLA determination relies on sequencing of the RNA associated with each HLA gene, accuracy is heavily influenced by transcription. The transcript levels of each HLA gene can vary greatly between tissues—for example, class II expression is much higher in the blood than in the liver—making HLA calling more difficult to predict for certain gene and tissue combinations. Transcript levels can also vary for the same gene and tissue across individuals, influencing the success of HLA calling. The combination of alleles expressed in an individual can also influence HLA calling success. For example, in some samples there may be allelic expression imbalance, a situation in which one particular allele may be expressed at a significantly higher level than the partner allele. Additionally, some alleles may be rare and not included in the database or two combinations of different alleles have very similar nucleotide content. In these cases, the HLA call rates may suffer for a specific subset of HLA alleles. Finally, some algorithms are only limited to calling HLA types for a subset of genes and alleles, often through database design, and these algorithms will be limited in their ability to call HLA alleles. Of the tools evaluated, only HLAProfiler and HLAForest were able to provide > 99% call rates for all of the major HLA genes (HLA-A, HLA-B, HLA-C, HLA-DP, HLA-DM, HLA-DO, HLA-DQ, and HLA-DR) (Additional file 2: Table S5). Of the two tools, HLAProfiler had slightly higher call rates for some important genes, such as MICA, MICB, TAP1, and TAP2 (99–100 vs. 42–96%), while HLAForest had better rates for HLA-H (25 vs. 100%) and many pseudo genes (e.g., J, K, L).

### TruSight HLA typing resolved discrepancies and increased HLAProfiler accuracy

Resolving HLA types using Sanger sequencing, while very accurate, is not perfect. For this reason, the true state of the sample is unclear whenever RNA sequencing results and Sanger results disagree. In this scenario, a third highly accurate technology can be used as the arbitrator. This approach will yield an improved understanding of the true state of the sample, provided that this technology has biases and errors that are reasonably independent of the first two. We attempted this arbitration using Illumina’s TruSight HLA panel. We believe that this method yields results that are sufficiently different from RNA sequencing because 1) it uses DNA as the genomic material; 2) it utilizes a targeted, rather than whole-transcriptome, approach; and 3) the software uses a different algorithm than any of the other tools. Including all samples discordant between any caller and the gold standard was cost prohibitive; therefore, we chose to investigate only discordances from the top two performing algorithms.

We collected all samples that had any discordance between either HLAProfiler or OptiType and Sanger sequencing, which amounted to 35 samples and 39 discordant calls. We sequenced 34 of these samples with Illumina’s TruSight HLA panel (Additional file 2: Table S6), in addition to two samples with only one-field HLA precision, previously available for one of the genes. After replacing the Sanger calls with the TruSight HLA calls for these 40 (38 discordant, two one-field precision only) allele calls in the truth data, performance metrics were recalculated (Fig. 2b; Additional file 2: Table S4). Using this updated truth, HLAProfiler had 99.9, 99.0, and 99.6% accuracy for HLA-A, HLA-B, and HLA-C, while OptiType had 99.6, 99.4, and 100% accuracy. In addition, HLAProfiler had 99.3 and 99.9% accuracy for DRB1 and DQB1. Finally, we used this updated truth to estimate the accuracy of the Sanger sequencing calls for these samples, which was found to be 99.7, 99.1, and 99.3% for HLA-A, HLA-B, and HLA-C and 99.0 and 99.4% for DQB1 and DRB1. For every gene except HLA-B, HLAProfiler produced a more accurate result than Sanger sequencing for this dataset.

### New analytical methods find disease alleles missed by gold standard technologies

**Fig. 3:**
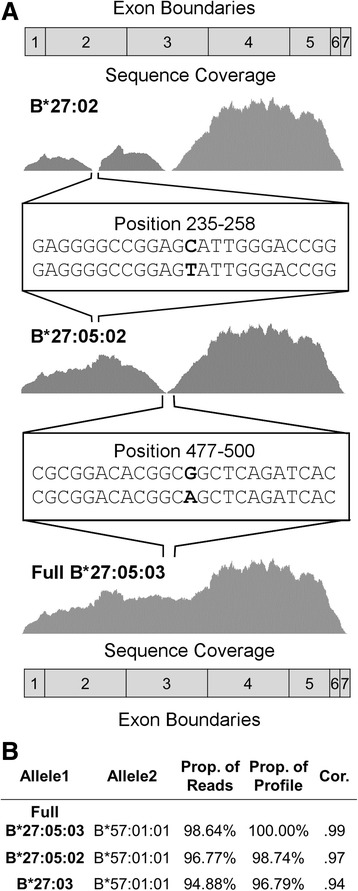
HLAProfiler correctly identifies the disease-associated B*27 allele incorrectly called by the gold standard. **a** Sequence coverage of RNA-seq data from NA11840 when aligned to B*27:03 (gold standard call), B*27:05:02 (identified by RNA-seq algorithms), and B*27:05:03 (full sequence predicted by HLAProfiler with allele refinement, and allele confirmed by TruSight HLA). Exon boundaries relative to the allele and differences between the alleles responsible for dips in coverage are also noted. **b** HLAProfiler generated comparison statistics of the three alleles, indicating the proportion of observed reads accounted for by the profile, the proportion of the profile accounted for by observed reads, and the correlation between the observed reads and the profile

In the Geuvadis data, we observed that all six callers evaluated failed to predict one of the B gene alleles in the gold standard reference for sample NA11840 (Fig. 3). While the gold standard lists the sample two-field genotype as B*27:03 and B*57:01, each of the callers correctly predicted (based on TruSight HLA) two-field types of B*57:01 and B*27:05. Without the allele-refinement step, HLAProfiler predicts B*27:05:02, which differs from B*27:03 at a single position in the reference sequence. Sequence coverage of the observed reads at this position supports B*27:05:02 but also shows a gap in coverage at position 489. Sequence reads at this position support a G < A change in the reference for B*27:05:02. This change corresponds to the sequence of B*27:05:03, which is partial and contains only exons 2 and 3 in the reference database. Running HLAProfiler with allele refinement correctly predicts B*27:05:03 as the top allele, and orthogonal typing using TruSight HLA confirmed this genotype. This level of accuracy is particularly important in this case. The HLA type B*27:05 is strongly associated with the disease ankylosing spondylitis, while the association of this disease with B*27:03 is less clear [35, 36]. In this case, and two others, Sanger sequencing inaccurately identified the HLA types, which may have led to a misdiagnosis of the patient. This highlights the challenge of using a database that comprises mostly partial alleles (87.8% do not contain the full protein sequence) and some complete alleles to identify HLA types using NGS data. These results additionally underscore the need for allele refinement to identify gaps in coverage caused by mismatches between observed reads and the reference sequence. Interestingly, two other samples, NA11832 and NA12005, also had B*27:05 as the correct allele but had different calls reported by Sanger (the allele group B*27:03/27:51/27:52/27:09 for NA11832 and B*27:03/27:52/27:09 for NA12005).

### HLAProfiler successfully identifies novel and partial alleles

With the B*27:05:03 allele in mind, we simulated an RNA-seq dataset to evaluate the ability of HLAProfiler to correctly identify the two-field HLA call or the protein sequence when only a partial sequence is available in the reference. We leveraged updates in the IMGT/IPD HLA reference (v3.26.0) released after the HLAProfiler database was created (v3.24.0) to identify alleles with a partial or incomplete reference sequence in the HLAProfiler database and a completed sequence in the more recent release. Using the rare simulation samples as a backbone, we randomly swapped the rare allele with one of these updated alleles per sample and simulated new RNA-seq data using the updated and complete sequence (Table 1). Using allele refinement, HLAProfiler correctly identified the exact full sequence for 67% of the samples and the two-field precision for an additional 18% of the samples, for a total of 85% with enough information to generate the correct protein sequence. While other callers were able to correctly identify the allele group (one-field precision) in most cases, they were unable to identify the protein (two-field) with the same accuracy. Of these, PHLAT had the highest two-field precision at 46%.

**Table 1:** Two-field accuracy and exact sequence matching for novel and partial alleles

	HLAProfiler	OptiType	seq2hla	HLAForest	HLAminer	PHLAT
Novel alleles*						
Sequence identified	63%	-	-	-	-	-
Sequence identified or two-field accuracy	68%	-	-	-	-	-
One-field accuracy	97%	15%	17%	19%	37%	96%
Two-field accuracy	25%	4%	4%	3%	10%	26%
Partial alleles						
Sequence identified	67%	-	-	-	-	-
Sequence identified or two-field accuracy	85%	-	-	-	-	-
One-field accuracy	97%	95%	94%	19%	72%	97%
Two-field accuracy	85%	41%	21%	3%	23%	46%

Results are based on two sets of simulated data for 100 samples, each having exactly one partial allele or one novel allele. Accuracy for novel alleles is defined as identification of the exact sequence, one-field accuracy, or two-field accuracy

*In the case of novel alleles, the correct protein sequence can be identified without correctly identifying one- or two-field precision, or one- or two-field precision can be identified while missing the exact protein sequence

Similar to the partial alleles, we also leveraged the updated database to evaluate the ability of HLAProfiler to identify novel alleles using the sequence data. We simulated novel allele sequences by replacing one allele per sample in the rare allele simulation with an allele present in the updated reference but not in the database reference (Table 1). After allele refinement, HLAProfiler predicted a novel allele sequence for 75 (75%) of the samples. For 63 (63%) of these samples the predicted sequence matched exactly to the sequence in the updated database (edit distance = 0). Combined with an additional 5% of alleles for which HLAProfiler was unable to identify the novel sequence but identified the correct two-field precision this represents a total of 68% of novel alleles with enough information to accurately identify the protein sequence. For the remaining 12 samples with incorrectly predicted novel sequences and 25 samples without predicted novel sequences the median edit distance between the predicted allele and the truth allele was 2 (Additional file 2: Table S7).

For comparison with other callers, we also determined the number of novel alleles correctly identified at two-field precision without allele refinement. Of the other callers, PHLAT had the highest two-field accuracy in novel alleles (26%). Of the 100 samples with a novel allele simulated using the updated database, only 36 contained a novel allele that shared two-field precision with an allele in the original database, highlighting the challenge of identifying the correct two-field precision of novel alleles. It is important to note that because of the missing two-field precisions in the database, the two-field precision of the base allele (reported by HLAProfiler) used to predict the correct protein sequence was often incorrect. Overall, HLAProfiler’s allele refinement algorithm is an important step in overcoming the challenges of predicting the correct protein group of partial and novel alleles. TruSight HLA also predicted the presence of novel alleles. HLAProfiler correctly predicted the sequences of all three novel alleles identified by TruSight HLA (Additional file 1: Table S8).

### HLAProfiler can be expanded to call KIR types

While the underlying database of *k*-mer profiles is specific to HLA, the algorithms inherent to HLAProfiler are more general and can be expanded to other genes. As a proof of concept, we applied HLAProfiler as-is to KIR genes, making no effort to optimize the code or tune parameters. For this, we created a KIR gene *k*-mer reference profile from IMGT and then randomly simulated types for 17 different KIR genes in ten samples (Additional file 1: Table S9). Using *k*-mer profiles for the KIR genes, HLAProfiler correctly identified > 90% of alleles for 12 of the 17 genes (70.6%). Two of the remaining four genes had greater than 65% accuracy. The final two genes, KIR2DL5A and KIR2DL5B, had 40 and 50% accuracy, respectively. Greatly reduced sequence depth after filtering, caused by high homology between these two genes, is the likely cause of the reduced accuracy for these two genes (Additional file 1: Figure S4). This proof of concept illustrates the flexibility of HLAProfiler in identifying alleles present from other immune-related genes, and optimizing HLAProfiler for KIR typing will undoubtedly yield much improved performance.

## Discussion

Accurate and precise HLA typing is critical in a variety of medical applications, such as organ transplantation, drug safety, disease susceptibility, and neoantigen prediction. RNA sequencing is an important source of transcriptome-wide gene detection and quantification and a promising source of HLA calls. Leveraging RNA-seq data for accurate HLA typing can not only reduce the cost, time, and input material requirements of HLA typing but also limit the need for specialized and separate laboratory protocols and reagents. Current algorithms have been limited in their ability to accurately predict HLA types from RNA-seq data in a wide range of HLA genes.

We have presented HLAProfiler, a *k-*mer profiling tool for accurately identifying the types of a variety of HLA genes in RNA-seq data. Our algorithm utilizes *k*-mer filtering, *k*-mer matching and competitive sequence matching and does not rely on traditional alignment, phasing or assembly tools. HLAProfiler was able to correctly identify HLA types with 100% accuracy at exact-allele precision in simulated RNA-seq data. These data were based on HLA types from 109 individuals, guaranteeing that the allele combinations exist in nature and are biologically relevant. This accuracy at such a high precision level represents a significant advancement over existing methods, most of which report only two-field accuracy.

HLAProfiler also performed well with RNA-seq data generated from lymphoblastoid cells. HLAProfiler called HLA alleles with > 98% concordance to gold standard typing at two-field precision in both class I and II alleles. The only tool with comparable performance, OptiType, also identified alleles with > 98% concordance but is limited to class I alleles. In the case of discordant calls, observed sequence reads often supported HLAProfiler calls rather than the gold standard. Orthogonal typing using Illumina’s TruSight HLA kit resolved these discrepancies and improved HLAProfiler concordance to greater than 99% for all genes.

HLAProfiler represents significant increases over existing methodologies when handling rare alleles, novel alleles, and alleles with partial reference sequences. When creating the reference *k*-mer profile database, HLAProfiler uses all available reference sequences and does not discriminate against rare alleles. This allows HLAProfiler to outperform all other callers, including OptiType, when calling rare alleles. HLAProfiler also includes partial reference allele sequences in the database. The percentage of alleles with a partial reference sequence in IMGT/HLA is staggering and, as demonstrated with NA11840, having partial allele calls can negatively impact allele calling. By identifying differences between the observed data and the initial prediction, HLAProfiler can correct these incorrect predictions due to partial alleles and identify the full allele sequence.

In addition, HLAProfiler can identify the presence of novel alleles. Identifying the presence of a novel HLA allele in an individual is important, especially in cases when the resulting protein sequence differs from the predicted allele. HLAProfiler can not only identify the presence of novel alleles but also predict the complete protein sequence over 60% of the time. Novel alleles are especially difficult to type correctly, resulting in multiple novel alleles being predicted by TruSight HLA in samples with discordant HLA types. In these three samples, HLAProfiler’s novel allele predictions matched exactly with the TruSight HLA prediction. In both the simulated and biological data, HLAProfiler successfully predicted novel HLA transcripts in RNA-seq data, which none of the other evaluated tools were able to do.

Despite the success of HLAProfiler in calling HLA alleles, HLA calling in RNA-seq data has limitations. Mainly, the technique is limited by the expression level of the HLA genes in the sample being evaluated. HLA calling, especially for class II genes, in tissues with decreased HLA expression will be more difficult than in whole blood or peripheral blood mononuclear cells, where the HLA genes are highly expressed. Additionally, HLAProfiler is sensitive to the coverage of the HLA genes, as demonstrated by the slight decrease in performance in downsampled reads. Samples with degraded RNA, such as formalin-fixed paraffin-embedded samples, where coverage across the genes is less uniform, might prove to be especially problematic. Improving both laboratory procedures and algorithms to increase the sequencing depth and coverage of HLA genes is an area of active research.

Our work offers a prime example of the utility of calling HLA alleles using RNA-seq data. One of the strongest putative associations between MHC allele and disease is the link between the B*27 antigen and AS, with approximately 90% of AS patients being B*27-positive [35]. For three samples, Sanger sequencing provided an HLA type of B*27:03, while RNA-seq gave a B*27:05 result, which was confirmed with targeted DNA sequencing. Although B*27:05 shows a clear association with AS, the association with B*27:03 is unclear [36]. This example illustrates how new analytical methods can provide accurate calls in lieu of traditional gold standard methods.

Overall, HLAProfiler offers a simple, flexible, and user-friendly approach to calling HLA alleles in RNA-seq data. We have also shown that HLAProfiler can be easily adapted to identify the alleles of other classes of genes, such as KIR genes, provided that the genes do not have extreme homology with other transcripts, the alleles for the gene are well curated, and only two allele states (e.g., germline diploid) are expressed.

## Conclusions

Precise and accurate identification of HLA alleles in RNA-seq data is difficult. Using RNA-seq data, we have demonstrated that HLAProfiler can accurately identify common and well documented alleles as well as rare alleles for class I and II HLA genes. Additionally, HLAProfiler utilizes the observed reads to detect when novel alleles are present or when the reference allele is incomplete and output the correct coding sequence for the allele. Finally, HLAProfiler successfully identified alleles for KIR genes, which are also important to immune response. Using HLAProfiler to reliably type HLA, and other gene classes in RNA-seq data will further increase the utility of these data and open the door to important biological discoveries without an increase in finite resources such as time and cost.

## Availability and requirements

Project name: HLAProfiler

Project home page: https://expressionanalysis.github.io/HLAProfiler/

Archived version: v1.0.5

Operating system: Linux, Unix

Programming language: Perl

Other requirements: EA-modified Kraken, Jellyfish

License: Custom

Any restrictions to use by non-academics: The software is limited to non-commercial use.

## Additional files

Additional file 1: Tables and Figure. **Table S1.** Counts for each population of real samples used in the current study. **Table S2.** Number of alleles available and used for each type of simulated data set. **Table S8.** Novel alleles identified by TruSight HLA for 33 HapMap samples having discordances between Sanger sequencing and RNA sequencing. **Table S9.** Accuracy of KIR genotyping at two-field precision. **Figure S1.** Representative HLA taxonomy. **Figure S2.** Run times of HLA calling software. **Figure S3.** Accuracy of HLAProfiler at low number of reads. **Figure S4.** Sequencing read count and KIR genotyping accuracy. (PDF 584 kb)

Additional file 2: Table S3–S7. **Table S3.** Accuracy of HLA calling in simulated data.** Table S4. **Concordance of HLA calling with "gold standard" in Guevadis data. ** Table S5. **Call rate for all methods across all genes for Guevadis data set. **Table S6. **Discrepant calls resolved with TruSight HLA typing. **Table S7.** Comparison of predicted sequence to the truth sequence in novel allele dataset. (XLSX 67 kb)
