# Supplementary material for: HLAProfiler utilizes k-mer profiles to improve HLA calling accuracy for rare and common alleles in RNA-seq data
Source: Genome Med. 2017 Sep 27;9:86. doi: 10.1186/s13073-017-0473-6 (PMC5618726; doi:10.1186/s13073-017-0473-6)
Supplement: Supplementary file 1 — Table S1. Counts for each population of real samples used in the current study. Table S2. Number of alleles available and used for each type of simulated data set. Table S8. Novel alleles identified by TruSight HLA for 33 HapMap samples having discordances between Sanger sequencing and RNA sequencing. Table S9. Accuracy of KIR genotyping at two-field precision. Figure S1. Representative HLA taxonomy. Figure S2. Run times of HLA calling software. Figure S3. Accuracy of HLAProfiler at low number of reads. Figure S4. Sequencing read count and KIR genotyping accuracy. (PDF 584 kb) [file 13073_2017_473_MOESM1_ESM.pdf]

SUPPLEMENTAL FIGURES AND TABLES

HLAProfiler utilizes *k*-mer profiles to improve HLA calling accuracy for rare and common alleles in RNA-seq data

Martin L. Buchkovich[1 †*], Chad. C. Brown[1 †], Kimberly Robasky[1], Shengjie Chai[2,3], Sharon Westfall[1], Benjamin G. Vincent[2,3,4], Eric T. Weimer[5], Jason G. Powers[1]

[†]Contributed equally

*Correspondence: martin.buchkovich@q2labsolutions.com

[1]Translational Genomics Department, Q[2] Solutions | EA Genomics, a Quintile Quest Joint Venture, Morrisville, NC, 27560, USA

[2]Lineberger Comprehensive Cancer Center, University of North Carolina at Chapel Hill, Chapel Hill, NC, 27599, USA

[3]Curriculum in Bioinformatics and Computational Biology, University of North Carolina at Chapel Hill, Chapel Hill, NC, 27599, USA

[4]Division of Hematology/Oncology, Department of Medicine, University of North Carolina at Chapel Hill, Chapel Hill, NC, 27599, USA

[5]Department of Pathology and Laboratory Medicine, University of North Carolina at Chapel Hill, Chapel Hill, NC, 27599, USA

Population	CEPH	FIN	GBR	TSI	YRI
Count	50	95	90	85	38

Table S1: Counts for each population of real samples used in the current study. Here, CEPH = Utah residents with mostly European ancestry, FIN = Finnish, GBR = British, TSI = Italy, YRI = Nigerian

Gene	Rare Alleles	Partial Alleles	Novel Alleles	Rare Sim	Updated Sim	Novel Sim
A	137	10	5	200	18	6
B	254	13	9	200	17	8
C	240	42	81	200	60	86
DMA	2	0	0	200	0	0
DMB	3	0	0	200	0	0
DOA	7	0	0	200	0	0
DOB	6	0	0	200	0	0
DPA1	5	0	0	200	0	0
DPB1	7	0	0	200	0	0
DQA1	14	0	0	200	0	0
DQB1	50	1	0	200	2	0
DRA	4	0	0	200	0	0
DRB1	19	0	0	200	0	0
E	7	1	0	200	3	0
F	5	0	0	200	0	0
G	17	0	0	200	0	0
MICA	13	0	0	200	0	0
MICB	6	0	0	200	0	0
TAP1	7	0	0	200	0	0
TAP2	5	0	0	200	0	0

Table S2*:* Number of alleles available and used for each type of simulated data set. Column "Rare Alleles" describes how many alleles had full sequences available and also were not previously reported as being "common" or "well-documented". Column "Partial Alleles" describes the number of alleles having partial sequences in IMGT/HLA version 3.24.0 and full sequences in version 3.26.0. Novel Alleles are how many alleles full sequences exist in 3.26.0, but did not exist in 3.24.0. The "Sim" columns describe how many alleles were simulated in each dataset.

Sample	Gene	Exon	Closest allele	Nucleotide change	Amino acid change	Detected by HLAProfiler
NA18505	HLA-A	6	26:01	bpT2829C	No	Yes
NA19093	DPA1	4	03:01	bpG5418C	No	Yes
NA19130	DQB1	4	04:02	bpC6300T	No	Yes

Table S8 Novel alleles identified by TruSight HLA for 33 HapMap samples having discordances between Sanger sequencing and RNA sequencing

KIR2DL1	95.00%
KIR2DL2	75.00%
KIR2DL3	95.00%
KIR2DL4	90.00%
KIR2DL5A	40.00%
KIR2DL5B	50.00%
KIR2DP1	95.00%
KIR2DS1	90.00%
KIR2DS2	90.00%
KIR2DS3	100.00%
KIR2DS4	95.00%
KIR2DS5	90.00%
KIR3DL1	95.00%
KIR3DL2	90.00%
KIR3DL3	95.00%
KIR3DP1	80.00%
KIR3DS1	65.00%

Table S9: Accuracy of KIR genotyping at two-field precision.

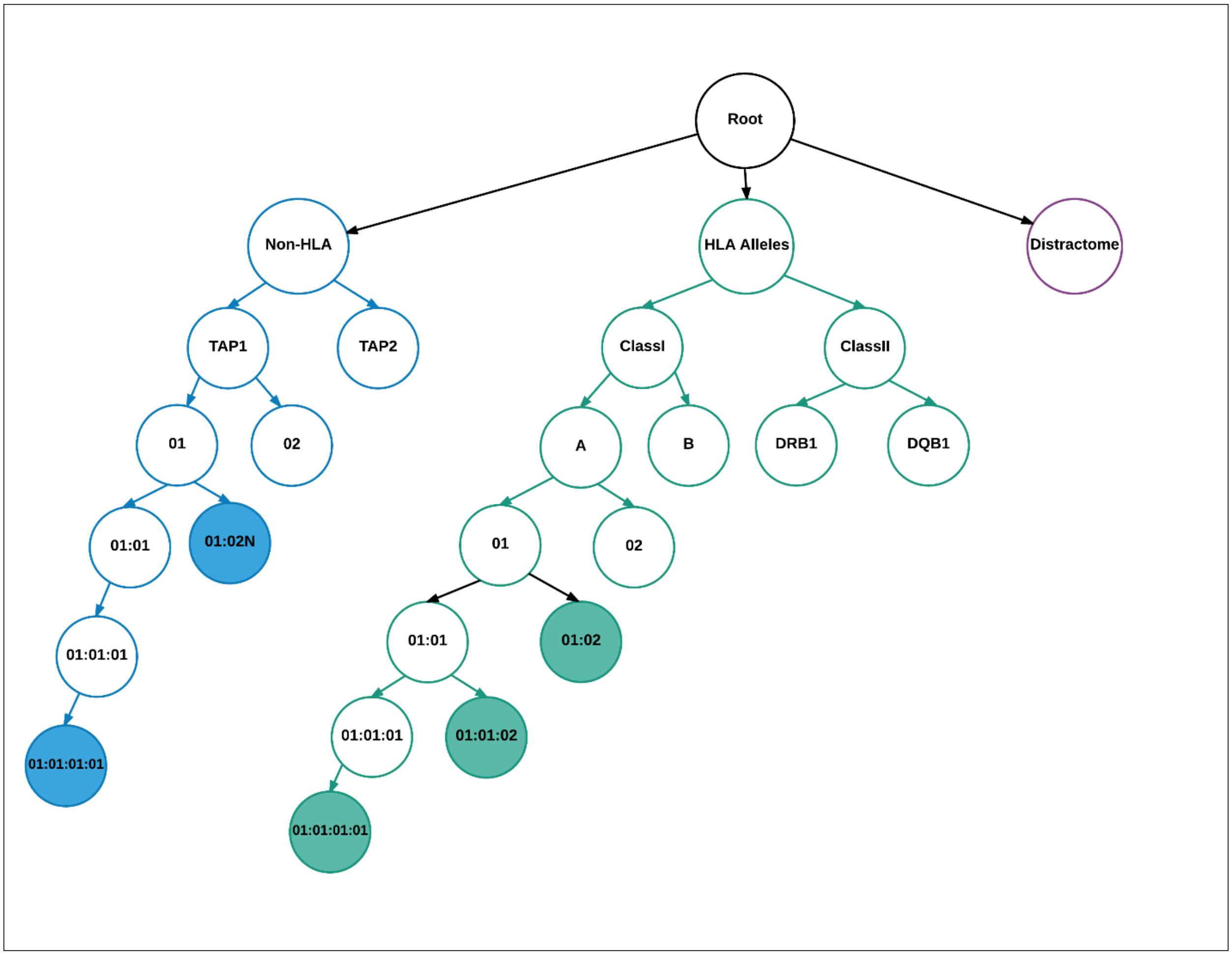

Figure S1. Representative HLA taxonomy. This simple HLA taxonomy represents the more complex, full HLA taxonomy used with the Kraken taxonomic classification software. HLA alleles are divided into a tree based on class, gene, and the precision level of the allele. Genes in the HLA region not falling into ClassI and ClassII are classified in a Non-HLA gene class. The distractome represents all genes not found in the HLA region. Leaf nodes (filled circles) represent actual HLA alleles while other nodes (open circles) represent common ancestors of the allele.

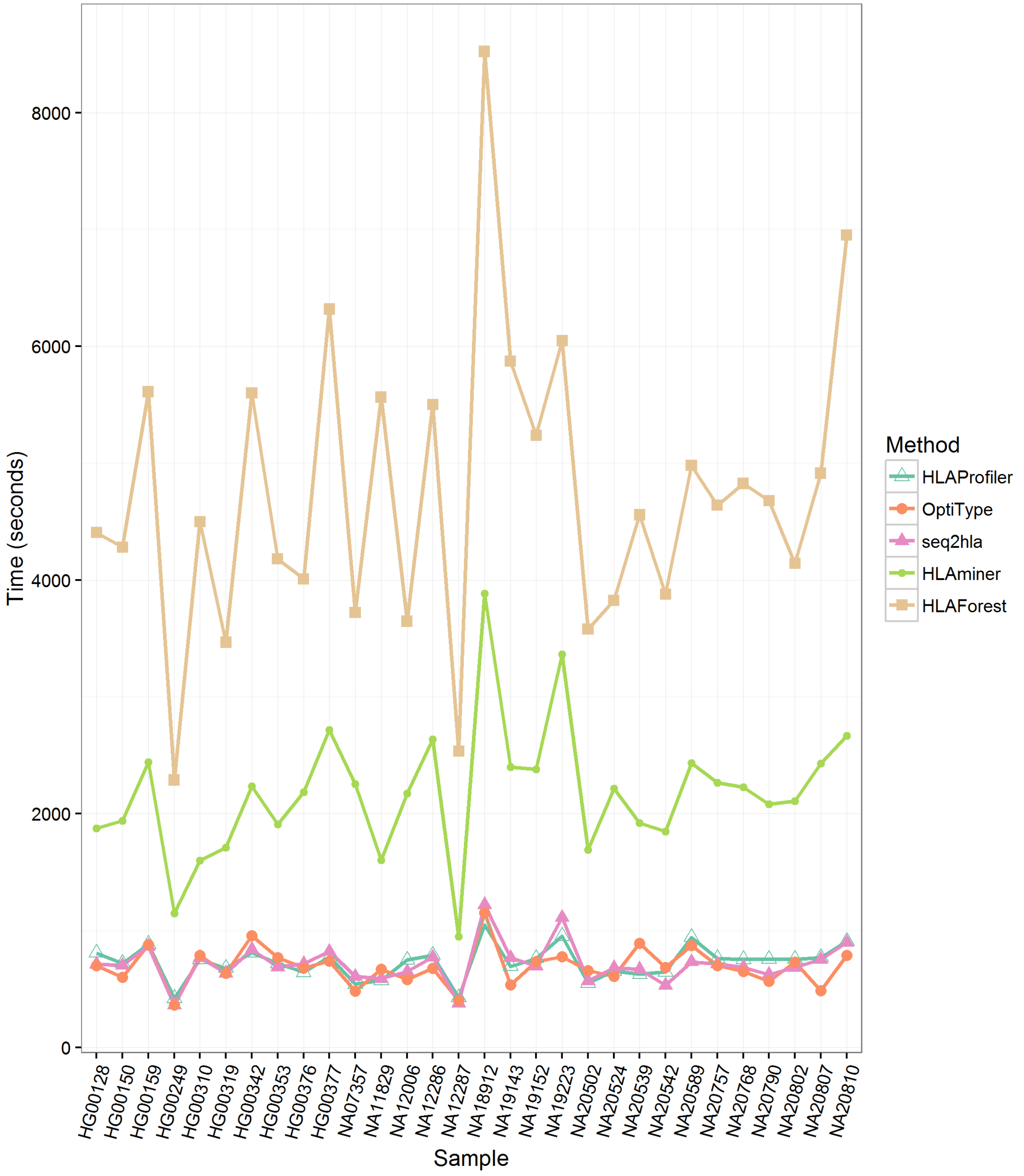

Figure S2: Run times of HLA calling software. Time required to call HLA types in 30 samples randomly selected from the Geuvadis dataset. Each tool was run on the same linux system using 8 threads. Due to licensing restrictions Phlat could not be run on the same system as the other tools and was excluded from this comparison.

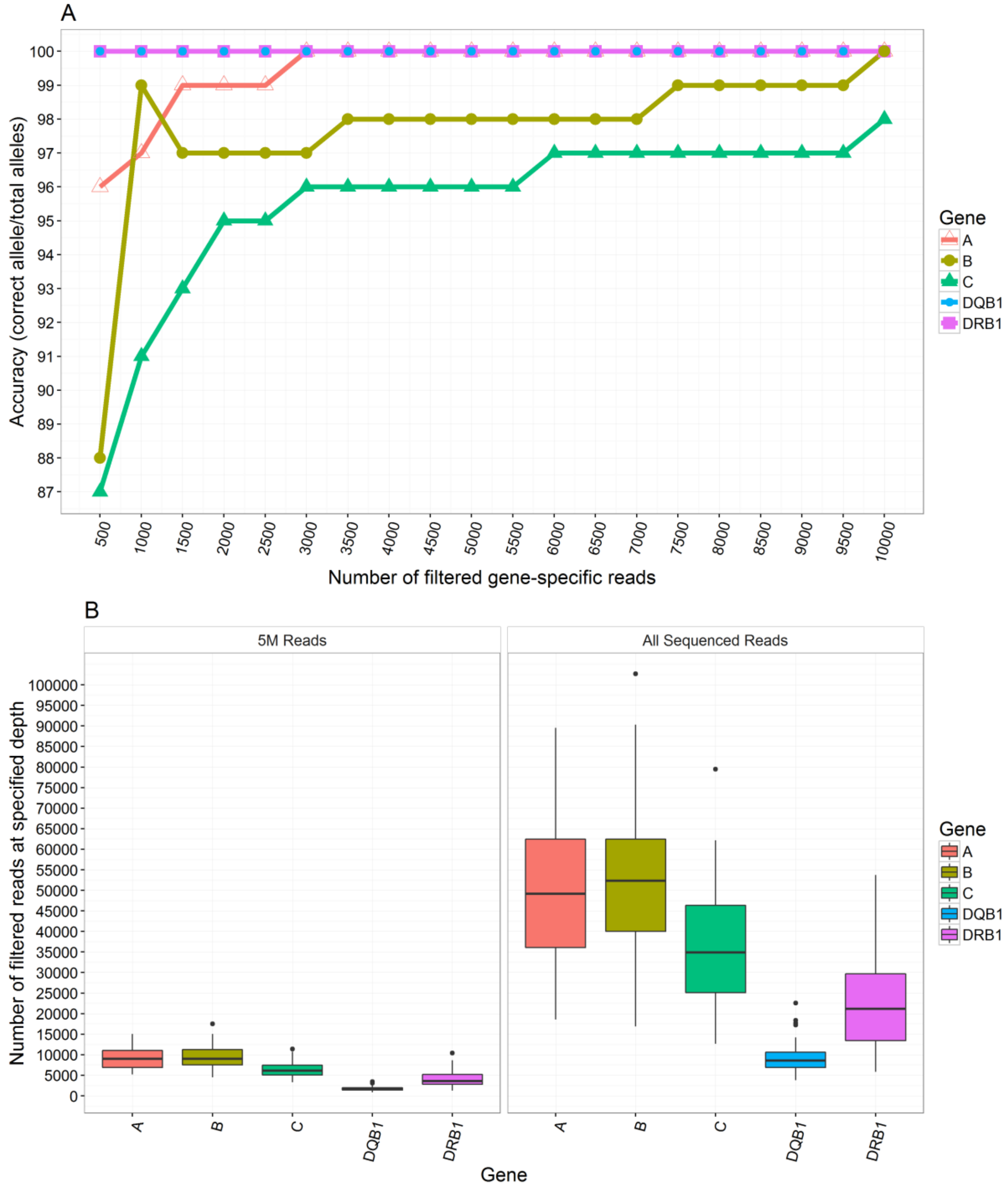

Figure S3: Accuracy of HLAProfiler at low number of reads. 50 Geuvadis samples with all alleles correctly predicted were randomly selected. Filtered reads specific to each gene were downsampled and HLAProfiler run on the downsampled reads. (A) HLAProfiler accuracy at given read depths.(B) The distribution of the number of filtered reads for each gene at a 5M total read depth and when using all sequenced reads (total depths differ).

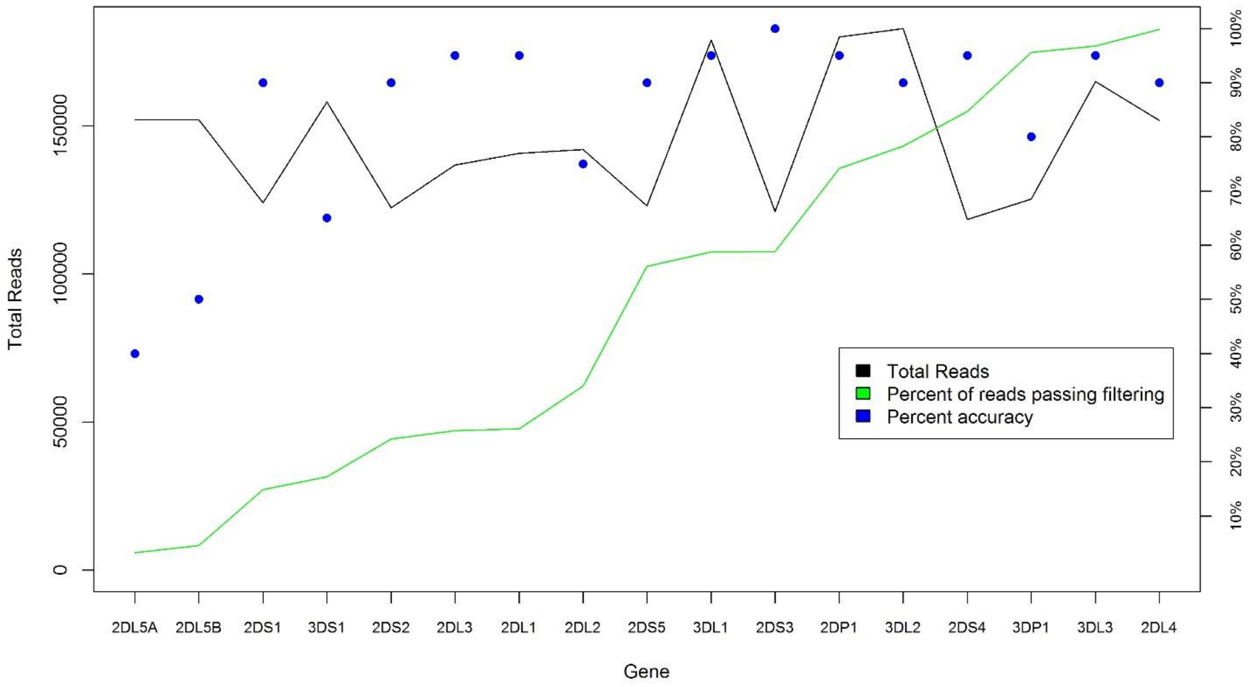

Figure S4: Sequencing read count and KIR genotyping accuracy. KIR genotyping accuracy (blue circle) for each KIR gene compared the total number of reads (black line) simulated for that gene, and the number of reads included in the gene sequences after filtering (green line).
